# Translating scores into meaning: a co-produced forum for patient-reported outcome measure interpretation in pediatric congenital heart disease

**DOI:** 10.1186/s40900-026-00981-7

**Published:** 2026-09-28

**Authors:** Jana Willems, Anna Eyrich, Jens Terjung, Alina Stricker, Vera König, Alienor Ringwald, Petra Huth, Brigitte Stiller, Thorsten Langer, Christoph Zürn

**Affiliations:** 1 Department of Neuropediatrics and Muscle Disorders, Faculty of Medicine, Medical Center, University of Freiburg, Freiburg, Germany; 2 Department of Congenital Heart Defects and Pediatric Cardiology, University Heart Center Freiburg, Medical Center-University of Freiburg, Faculty of Medicine, University of Freiburg, Freiburg, Germany; 3 Herzklopfen e.V, Freiburg, Germany

**Keywords:** Patient involvement, Co-production, Integrated knowledge translation, Patient-reported outcome measures, Pediatric congenital heart disease

## Abstract

**Background:**

Patient and public involvement (PPI) and co-production are intended to increase the relevance and uptake of health research, yet structured ways of jointly interpreting patient-reported outcome measure (PROM) data in real time remain limited. We developed and tested a PROM-anchored participatory forum within a longitudinal pediatric congenital heart disease (CHD) study.

**Methods:**

The forum was co-developed and co-delivered by a patient organization and a university hospital as an explicit integrated knowledge translation (iKT) episode. Interim Pediatric Quality of Life Inventory™ Cardiac Module (PedsQL CM) results were presented in a plain-language life-course graphic and discussed in two rounds entailing small-group reflection and prioritization. Data sources included facilitator notes, plenary clusters, poll outputs, post-forum evaluation responses, and baseline PROM patterns. We took a framework-guided thematic approach and adapted joint displays to integrate qualitative and quantitative data.

**Results:**

Twenty families participated. The format enabled participants to contextualize aggregated PROM findings in relation to everyday life, and to identify where quantitative patterns resonated with lived experience and where additional nuance was needed. The forum produced co-produced interpretation memos, a prioritized action list, an implementation and adaptation package pack, and the family-facing dissemination figure called the “Quality-of-Life Tree”. Participants highlighted transition-sensitive concerns, emotional burden, care coordination, and the need for child-friendly explanations and identifiable points of contact. The process was perceived as comprehensible and useful.

**Conclusions:**

A PROM-anchored participatory forum can make interim outcome data usable before the end of data collection and translate them into shared interpretation, action planning, and family-friendly dissemination. The approach offers a transferable model for linking PROM measurement, participatory interpretation, and follow-up in pediatric care research.

**Trial registration:**

German Clinical Trials Register (DRKS), DRKS00028565. Registered on March 30, 2023.

**Supplementary Information:**

The online version contains supplementary material available at https://doi.org/10.1186/s40900-026-00981-7.

## Abstract (plain language summary)

Patient and family involvement is increasingly important in health research, but it is not always clear how families can be involved in making sense of questionnaire results. In this study, we created a forum where families of children with congenital heart disease, healthcare professionals, and a patient organization discussed early results from a health-related quality-of-life questionnaire for children and adolescents with congenital heart disease.

We presented the results in a visual format and asked participants to talk about what the findings meant to them in everyday life. The forum helped families explain where the results matched their experiences and where more context was needed. It also helped the group agree on the next steps, such as clearer information for families, better communication with hospitals and community services, and more support around transition points in family life.

The forum produced several practical outputs: short interpretation notes, a list of priorities, a package for others who may want to take the same approach, and a family-friendly summary figure called the “Quality-of-Life Tree”. Participants found the forum understandable and useful.

Our findings suggest that questionnaire results can help turn data into action and make research more useful for families and care teams. Furthermore, forum-based formats appear to provide a solid foundation for the collaborative interpretation of data from patient-reported outcome measures.

**Supplementary Information:**

The online version contains supplementary material available at https://doi.org/10.1186/s40900-026-00981-7.

## Introduction

Patient and public involvement (PPI) and co-production approaches aim to increase the relevance, legitimacy, and uptake of health research by involving patients, families, and other stakeholders throughout the research process [1–3]. A wide range of participatory formats has been described including advisory panels, co-design workshops, and town-hall-style forums, commonly used to identify priorities, articulate needs, or co-design services and interventions [4–6]. Systematic reviews indicate that different formats generate different outputs: consultative approaches often yield reports or recommendations, whereas higher levels of engagement such as co-design or research partnership are more likely to result in structural or process changes to services [5, 7]. Town-hall and forum-based formats have been used effectively for setting priorities and shaping the agenda [8, 9]; however, they have rarely been described as transferable mechanisms for the joint interpretation of patient-reported outcome measure (PROM) data.

PROMs are increasingly used in pediatric and chronic-care settings to assess health-related quality of life (HRQoL) and to support communication between patients, families, and clinicians. Evidence suggests that PROM feedback can improve symptom recognition, clinical communication, as well as documentation and, under certain conditions, patient outcomes including quality of life [10, 11]. Recent work in pediatric PROM implementation emphasizes that collecting patient-reported data alone is insufficient: findings must be meaningfully fed back into care and service planning (“if we ask, we must act”) [12]. Implementation research also suggests that PROM feedback is most effective when embedded within clear care pathways and quality improvement processes rather than remaining an isolated measurement activity [13]. In this context, PROM domain patterns can function as shared artefacts for deliberation, contextualization, and action planning.

Embedding the participatory interpretation of PROM data into the research workflow aligns with the principles of community-based participatory research (CBPR) and integrated knowledge translation (iKT), both of which conceptualize knowledge production as a collaborative process involving researchers, affected individuals, and practice partners throughout the research lifecycle [14, 15].

In this methods article, we describe a transferable PROM-anchored participatory forum co-developed and co-delivered by a patient organization and a university hospital, as an explicit iKT episode within a longitudinal study applying the Pediatric Quality of Life Inventory™ Cardiac Module (PedsQL CM) [16, 17]. Rather than using a forum to set research priorities before data collection, we positioned it during quantitative data collection leveraging aggregated PROM findings as a shared starting point for participatory interpretation and contextualization and for co-generating practice-oriented next steps. Unlike conventional stakeholder workshops, which commonly use experiential knowledge to identify priorities or co-design interventions, our forum used aggregated PROM findings as the starting point for jointly interpreting what the observed patterns might mean in everyday life and what actions they might warrant.

While PROM-based HRQoL assessments are increasingly used in pediatric CHD research [18], structured participatory interpretation processes embedded within longitudinal PROM studies remain uncommon. Specifically, we (a) describe the forum design and analytic approach, (b) situate the method within published evidence on patient engagement, PROM implementation, and iKT, and (c) present a transferable protocol for PROM-anchored participatory interpretation.

## Methods

### Methodological framing and study embedding

This article is a methods-focused contribution describing a transferable participatory procedure for PROM interpretation embedded within a longitudinal pediatric HRQoL study of children and adolescents with congenital heart disease (CHD) and their caregivers [17]. Accordingly, the present paper focuses on procedural outputs, process characteristics, and short post-forum evaluation data rather than on substantive HRQoL outcomes. The underlying study uses the Pediatric Quality of Life Inventory™ Cardiac Module (PedsQL CM) to assess self- and proxy-reported HRQoL [16]. Data are collected electronically at baseline and at three, six, nine, and 12 months. Our study protocol was approved by the University of Freiburg’s ethics committee (Reference No. 22-1451-S1).

The forum was conducted approximately 18 months after recruitment had commenced, while quantitative data collection was still ongoing. Because recruitment occurred continuously, forum participants were at different stages of the longitudinal assessment schedule. Baseline assessment was anchored to the inpatient admission for an intervention, either cardiac surgery or cardiac catheterization, which constituted an inclusion criterion [17]. The forum used aggregated baseline PedsQL findings available at that time as the basis for participatory interpretation; subsequent follow-up assessments continued independently as part of the longitudinal study.

### Characteristics of the underlying longitudinal study population

At the time of the forum, baseline data were available from 122 children and adolescents aged 2.0–17.3 years (mean 8.0 years, SD = 4.2); 48% were female and 52% male. Within this cohort, and according to Varni’s heart disease categorization [19], 26% had mild disease, 18% moderate disease, 24% disease with significant residua or need for further surgery, and 32% complex or severe disease, including single-ventricle physiology.

The baseline findings used for the forum indicated substantial heterogeneity in HRQoL across domains and age groups. Mean overall PedsQL CM scores were 61 (0–100 scale, higher values indicating fewer problems/better HRQoL), with particularly low scores observed for cardiac symptoms/physical strain and treatment anxiety. HRQoL scores generally decreased with increasing age.

### Design rationale and integrated knowledge translation framing

The forum was explicitly designed as a concentrated iKT episode within a broader, ongoing partnership between the research team and Herzklopfen e.V. during the ongoing study. It positioned patients, caregivers, clinicians, patient organization representatives, and researchers as interpretive partners rather than as information recipients across activities extending from agenda-setting to follow-up. Rather than treating PROM results as outputs to be disseminated unidirectionally, the forum conceptualized them as shared interpretive artefacts around which meaning, contextual relevance, and implications for practice could be jointly negotiated.

We conceptualized the forum as a methodological format defined by specifiedintegral to the design, delivery, and follow input, a structured deliberative sequence, and predefined output formats. Two methodological aims guided the forum design:

**Participatory data interpretation**: PROM domain trends served as structured prompts to elicit contextualized meanings, lived examples, and explanatory narratives from those with lived experience.

**Structured translation into practice**: The forum sought to co-produce, prioritize, and assign implementable actions, with clinical and patient/family advocacy partners explicitly mapping follow-up responsibilities during the same deliberative episode.

These aims align with co-production mechanisms described in the literature including developing shared understanding, identifying and addressing needs, and fostering ownership, trust, and accountability among stakeholders [5].

### Patient and public involvement

PPI was integral to the design, delivery, and follow-up of the forum. Herzklopfen e.V., a German parent-led association for families of children with CHD, acted as a full partner in this iKT episode. The organization’s chairperson and mother of a child with CHD played an active co-design role during regular preparatory meetings with the research team and an external moderator. An initial concept draft was discussed within Herzklopfen’s board, comprised of parents of children with CHD, and revisions were made iteratively based on their feedback.

Herzklopfen e.V. co-designed the recruitment materials including the flyer, invitation text, and dissemination channels, hosted the online event, managed registration, and played a central role during the forum. After the event, a structured debriefing and joint analysis session with Herzklopfen e.V. served to review findings and plan the following implementation steps. In line with engagement taxonomies, the involvement of Herzklopfen e.V. corresponded to a co-production level, as the organization contributed to agenda-setting, design decisions, recruitment strategy, delivery, interpretation, and follow-up planning. The completed GRIPP2 short-form checklist (Guidance for Reporting Involvement of Patients and the Public, version 2) is provided as Additional File 1.

### Participants and recruitment

Participating stakeholders included children/adolescents with CHD and their caregivers; representatives of Herzklopfen e.V., and clinical staff and research team members including a pediatric cardiologist, nurse, and psychosocial professionals. Clinical staff were primarily involved as small-group facilitators to enable deliberation.We recruited families via multiple channels: email lists and social media accounts of Herzklopfen e.V. and the university hospital, an official press release from the university hospital, announcements in official messenger and family-related activity groups of Herzklopfen e.V., a request for dissemination through other associations for children with heart conditions, an online registration link on the organization’s website, printed flyers, and personal outreach in outpatient and inpatient areas. Although recruitment via a parent-led organization may have preferentially engaged more active families, we also used clinic channels and did not require organizational membership. The invitation emphasized that no special expertise was required and that participation was free of charge. Participation in the study was not a prerequisite for attending the forum. All participants received written information about the forum including the planned use of anonymized notes for research and quality improvement, and provided digital informed consent; caregivers gave consent on behalf of adolescents under 18 years where required. To support the practical organization of age-appropriate breakout groups, limited participant information was collected during registration. This included broad age categories and sex of the children represented in participating families, as well as medication use. No detailed clinical information or specific CHD diagnoses were collected, both to minimize data collection burden and to avoid collecting potentially identifying clinical information in this relatively small participant group. These data were used solely for organizational purposes and were not intended as a systematic characterization of the forum sample.

### Forum structure

The 90-minute online forum via Webex followed a structured deliberative sequence designed to move from the shared interpretation of PROM results toward prioritizing and planning the next steps. Anonymous poll and survey exports generated during the session constituted an additional data stream for methodological analysis.

1. **Welcome and orientation** (10 minutes): Joint opening by the patient organization and research team, outlining aims, roles and ground rules. An initial chat-based icebreaker activated early participation and generated baseline interpretive frames.
2. **Plain-language presentation** (10 minutes): Clarifying the concept of HRQoL, outlining the longitudinal study design, and familiarizing participants with the questionnaire domains as well as visual presentation of interim PROM results using a plain-language, age-graded life-course graphic (see section “Plain-language presentation”).
3. **Small breakout groups I - reflection and sense-making** (20 minutes): The forum comprised four age-dependent breakout groups, each with five participants and two facilitators. Facilitated groups discussed the resonance between the PROM results and lived experience using structured prompts linked to PedsQL CM domains. Participants were asked in advance to indicate their child’s age so that small groups could be formed around age-appropriate topics. Each group was led by two trained facilitators. The lead facilitator guided the discussion and fostered a supportive atmosphere, while the co-facilitator summarized and documented the group’s findings and presented them during the subsequent plenary session. Small-group facilitators relied on a standardized semi-structured guide to ensure consistency across breakout sessions.
4. **Plenary synthesis I** (10 minutes): The external moderator clustered group feedback verbally. A word cloud poll posed the question “Where are things/What is currently most difficult in your everyday life?” supported cross-group comparison and visible prioritization.
5. **Small breakout groups II - solutions and priorities** (15 minutes): The same small groups generated concrete needs, potential quick wins, and longer-term proposals. Participants were explicitly asked to attribute proposals to responsibility domains such as clinic, patient organization, joint working group, or other.
6. **Plenary synthesis II - prioritization and commitments** (15 minutes): Final prioritization was carried out via guided live polling according to the question “What is the most important next step?”. Proposed actions were linked to preliminary responsibility assignments in real time, and commitments by the clinic and patient organization were documented.
7. **Closure and evaluation** (10 minutes): Participants completed a short evaluation via Webex forms consisting of three Likert items on comprehensibility, ability to contribute, and usefulness, as well as two open-ended reflection questions.

### Process facilitation and moderation

The forum used a two-level facilitation structure. An external professional moderator, who was independent of clinical care, the research team, and the patient organization, facilitated the plenary sessions and coordinated the overall process. The moderator’s role focused on process facilitation rather than content steering: structuring discussion, supporting accessible language, and aiming to foster balanced participation across stakeholder groups. He also conducted the polls and surveys during the plenary sessions.

The breakout discussions were facilitated by pairs of members of the clinical team. To reduce perceived barriers to participation and encourage families to speak openly, physicians were deliberately not assigned as facilitators. Instead, facilitation was provided by members of the psychosocial team of the pediatric cardiology unit, including psychologists, educators, and a chaplain. They were supported by two medical students from the study team: a doctoral student conducting study interviews and a student research assistant responsible for participant recruitment.

Facilitators received preparatory training on group processes and participatory facilitation, including recognizing and appropriately managing dominant contributions, explicitly inviting less vocal participants into the discussion, and avoiding overburdening participants. Before the breakout sessions, group ground rules were established, including allowing others to finish speaking and maintaining confidentiality within the group. Facilitators used a standardized semi-structured guide to support consistency across groups.

The external moderator was responsible for the plenary-level process, whereas the small-group facilitators supported the structured discussion and subsequently presented the groups’ findings during the plenary session. They did not contribute their own substantive positions to the group discussions or subsequent reporting; their role was to facilitate the process and represent the perspectives articulated within their respective groups.

This structure was intended to combine independent process moderation at the plenary level with experienced facilitation during the small groups and aimed to support balanced participation without assuming that professional–family power asymmetries were fully eliminated.

### Plain-language presentation

The forum did not provide study participants with feedback on their individual PROM scores or trajectories. Instead, we provided an aggregated, plain-language presentation of the baseline findings across PedsQL CM domains and age groups, based on the sample recruited up to that point. The purpose of the forum was therefore not to validate individual participants' PROM results, but to provide a participatory context for interpreting and contextualizing aggregated interim findings in light of families' lived experiences.

**Fig. 1:**
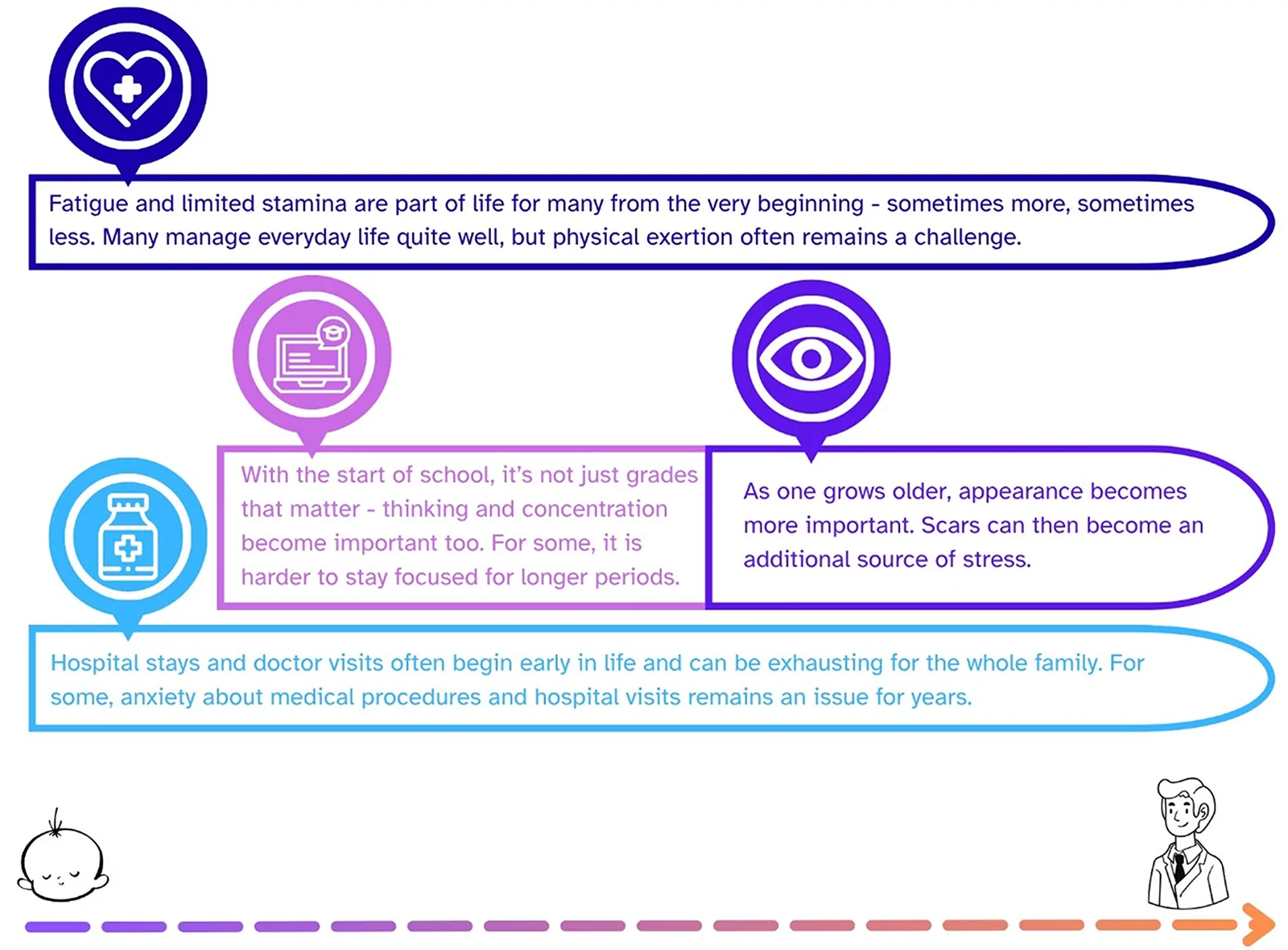
Life-course graphic used to present interim PedsQL CM findings in a plain-language, age-graded format. The graphic summarized baseline domain patterns across early childhood, preschool/school entry, primary school age, and adolescence and served as a shared prompt for participatory interpretation during the forum. Figure was generated using Canva software (Canva Pty Ltd, Sydney, Australia; 2026). Graphical icons were sourced directly from the Canva asset library

The visualization of results was embedded within a brief structured introduction clarifying the concept of HRQoL, outlining the longitudinal study design, and familiarizing participants with the questionnaire domains. Interim baseline findings from the PedsQL CM were translated into a plain-language, age-graded life-course graphic spanning early childhood, preschool/school entry, primary school age, and adolescence (Fig. 1). Rather than presenting detailed numeric subgroup tables, domain-level trends were synthesized into four thematic anchors: cardiac symptoms/physical strain, treatment-related anxiety, cognitive/school functioning, and body image/scars, positioned along an age continuum with selected anchors reflecting age-related variation observed in the aggregate baseline data.

This graphic served as a central interpretive prompt for the forum and functioned intentionally as a shared reference point for participatory contextualization of our interim findings. It was explicitly framed as an aggregated “aerial view” intended to stimulate comparison with lived experience rather than to represent any individual case. The graphic remained accessible to participants throughout the forum via a shared link, enabling them to refer back to it while sharing examples, clarifying meanings, or identifying gaps between the quantitative trends and everyday experience.

### Forum outputs and products

The forum generated the following output categories:

**Co-produced interpretation memos**

Short interpretation memos were developed from the small-group facilitators’ notes and the plenary synthesis. These memos combined baseline PedsQL CM patterns, forum narratives, and illustrative quotes where available. They documented where participants felt that trends accurately reflected lived experience, and where they perceived gaps or misalignments. The memos were organized around the age-graded life-course graphic and the themes emerging from the small-group discussions.

**Prioritized action list with responsibilities and time horizons**

Forum discussions led to a co-produced list of quick wins and longer-term goals clustered around relevant domains. For each proposed action, the list indicated where primary responsibility would lie, and outlined initial steps or avenues for further development.

**Forum implementation and adaptation package**

A forum script with timing, moderator and facilitator guides, evaluation template, and Webex poll templates were developed to support transferability and implementation and are available upon request.

**Dissemination of results**

**Fig. 2:**
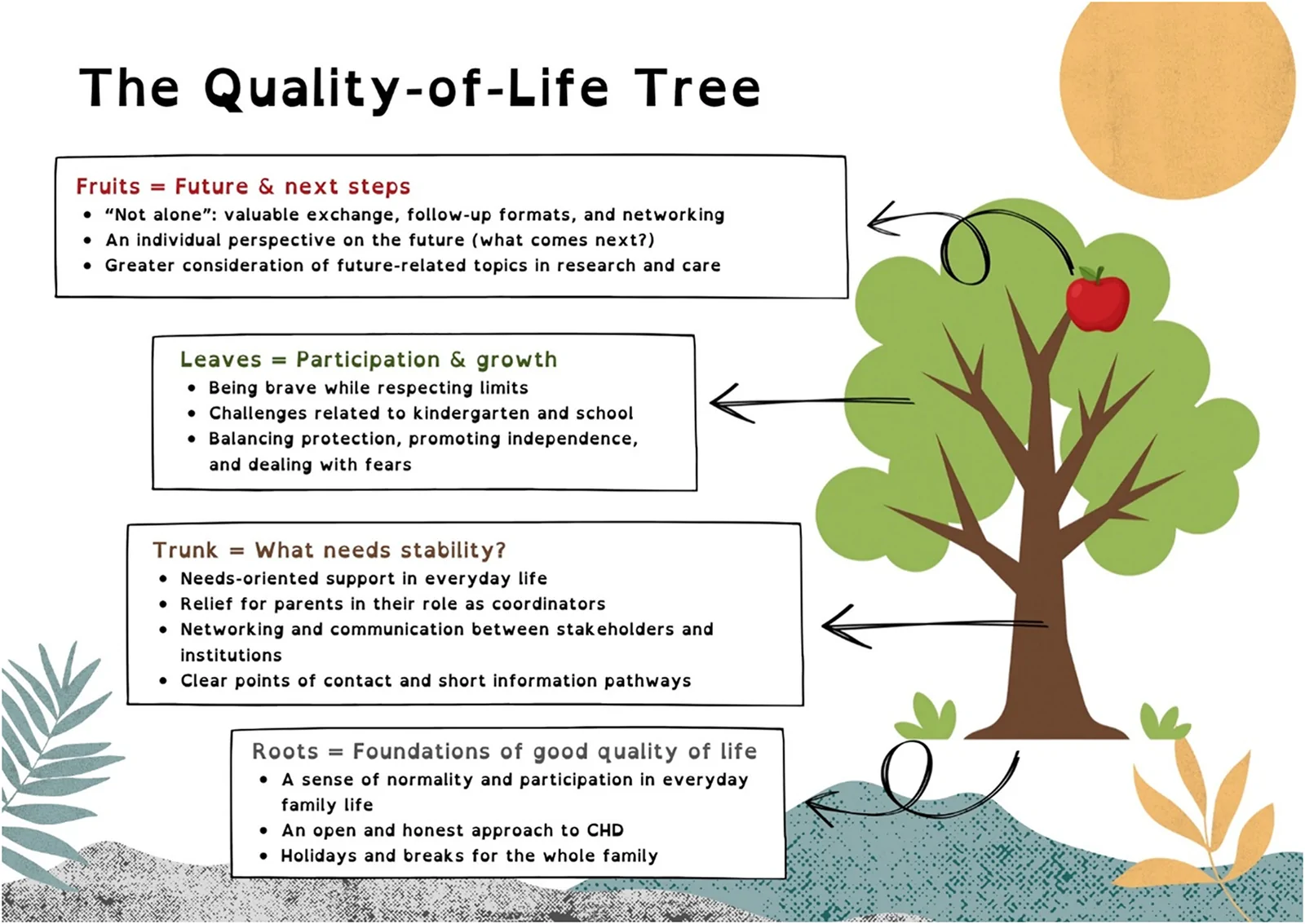
The “Quality-of-Life Tree” as a family-facing dissemination figure. The tree translated the forum’s co-produced interpretation into a visual summary organized around foundations of quality of life, stability needs, participation and growth, and future-oriented next steps. Figure was generated using Canva software (Canva Pty Ltd, Sydney, Australia; 2026). Graphical icons were sourced directly from the Canva asset library

To support a family-friendly return of the findings, we developed the “Quality-of-Life Tree” feedback figure, a metaphorical visual summary that condensed the main outputs into a single overview (Fig. 2). This figure translated the forum’s core messages into co-designed dissemination format by organizing them around foundational aspects of quality of life, stabilizing supports, changing challenges, and future-oriented next steps.

### Data sources

We used different data sources to support rapid participatory interpretation and actionable outputs:

- **Forum process and output data:** condensed small-group notes produced by small-group facilitators; facilitator synthesis notes summarizing breakout reports and plenary feedback; plenary poll and word cloud outputs
- **Post-forum evaluation:** short anonymous Webex survey with three Likert-items on comprehension, ability to contribute, and usefulness; two open-ended prompts: “What have you taken away?” and “Where should change happen?” (responses were visualized live using word clouds)
- **Quantitative PROM data:** baseline PedsQL CM aggregate domain trends by age band used to create the life-course graphic and to structure the forum prompts

Although the session and breakout discussions were not audio-recorded for ethical reasons and to protect participant well-being, methodological rigor was supported through triangulation across independent data streams, including facilitator notes, plenary clusters, and poll outputs, as well as cross-group comparison of emergent themes. All digital exports were anonymized by removing or redacting personal identifiers and stored on a secure institutional server.

### Analysis and integration with PROM data

We took a two-step qualitative-quantitative integration approach:

**Step 1 - Analysis of forum data**

We took a framework-guided thematic approach by relying on the forum structure and PedsQL CM domains as an initial deductive scaffold. Clustering was iterative, allowing inductive expansion for emerging need- and resource-oriented themes. This procedure was implemented in a rapid-compatible manner, relying on condensed notes and clustered summaries rather than full transcripts, while retaining transparency and rigor in analytic decision-making [20, 21]. In a post-forum debrief, the Herzklopfen e.V. chairperson and research team reviewed the emerging clusters, clarified meanings, and added missing points, thereby helping to align the interpretation more closely with the community perspective.

**Step 2 - Mixed-methods integration**

We constructed adapted joint displays that juxtaposed baseline PedsQL CM patterns by age group with forum-derived life-course interpretations and action proposals [22]. Integration was achieved through a PROM-anchored participatory interpretation process rather than a strict one-to-one matching of individual qualitative statements to quantitative scores. In this sense, the adapted joint displays functioned as an interpretive bridge and served to visualize how quantitative trends and qualitative interpretations converged, diverged, or complemented one another.Specifically, integration occurred at four levels:

- **Design integration:** PROM domains structured the breakout prompts and thematic focus.
- **Process integration:** The life-course graphic remained accessible throughout the forum, enabling participants to repeatedly refer back to and reinterpret the quantitative patterns and anchor their interpretations in the trends described.
- **Analytic integration:** The displays positioned aggregated quantitative patterns alongside forum-generated interpretations to enhance comparison and contextualization.
- **Output integration:** Action items were explicitly mapped back to relevant PROM domains and age-related patterns; the final thematic clustering and the “Quality-of-Life Tree” figure translated the forum-derived outputs into a format that could be shared with families and the patient organization.

## Results

As this is a methods-focused article, our results are reported primarily in terms of procedural feasibility, interpretive value, and co-produced outputs, thereby aligning our reporting with the core domain requirements of the GRIPP2 framework [20].

### Characteristics of families participating in the forum

Twenty families participated in the forum, including 21 parents/caregivers (13 female, 8 male) and 6 children/adolescents (2 female, 4 male). Across these families, 20 children were represented, ranging in age from 0 to 17 years. Their age distribution was as follows: 5 aged < 1 year, 5 aged 2–4 years, 2 aged 5–7 years, 2 aged 8–12 years, and 6 aged 13–17 years. This distribution refers to all children represented by the participating families, not only to the six children and adolescents who attended the forum. Information on medication use was available for 18 families: 14 reported medication use, 2 reported no medication use, and 2 provided no information. Of those 20 families, 17 were also represented in the longitudinal study.

### Method outcomes and interpretive value

The format enabled participants to contextualize the PedsQL CM findings in relation to their everyday life, and to identify where aggregate patterns resonated with lived experience and where additional nuance was needed. Families interpreted cognitive functioning not simply as an academic domain, but as a transition-sensitive area shaped by kindergarten and school entry, participation and social comparison, and the parental effort to balance protection with independence. Anxiety-related trends were interpreted in relation to fears around medical procedures, uncertainty about what comes next, and the everyday burden of anticipating future challenges. Physical strain and the need for rest were described as fluctuating with the surgical and recovery phases rather than remaining constant across age groups. The forum showed how broad PROM domains translated into lived experiences around topics that are not fully captured by the PedsQL CM, e.g., participation, transition points, emotional burden, and care coordination demands. The contextualization added a narrative frame to the quantitative domain trends that suggested potential points for a clinical response beyond what scores alone would imply. By situating the life-course graphic within lived experience, participants identified transition points in family life at which psychosocial check-ins and pre-procedural counseling may be particularly relevant. The adapted joint displays allowed forum-generated priorities to be read alongside the PROM trends, so that qualitative insights both illustrated the quantitative results and actively shaped how the findings were understood and translated into clinical and organizational implications. This approach aligns with mixed-methods integration principles by enhancing transparency, comparison, and interpretive depth in PROM-based implementation research [12, 13, 21].

### Outputs delivered

The forum outputs were consolidated into co-produced interpretation memos, a prioritized action list, an implementation and adaptation package, and a family-facing dissemination figure (“Quality-of-Life Tree”). The action list linked short-term priorities such as child-friendly pre-procedure information, and longer-term priorities such as improved communication between hospitals and community providers, to the relevant PedsQL CM domains. This produced a transparent path from measurement to implementation and made the proposed next steps legible to both families and professionals.

### Process evaluation

The short post-forum evaluation provided a brief assessment of participants’ perceptions of the process. We received 19 evaluation responses based on a 5-point Likert scale (1 = strongly disagree, 5 = strongly agree). Overall comprehensibility was rated highly (mean 4.7/5; 28% agreed and 72% strongly agreed that they understood the forum and its results). Participants also reported that they were able to contribute their perspectives (mean 4.4/5; 10% neither agreed nor disagreed, 37% agreed, and 53% strongly agreed) and perceived the forum as helpful and worthwhile (mean 4.4/5; 10% neither agreed nor disagreed, 37% agreed, and 53% strongly agreed).

Open-ended responses complemented these ratings and clustered around three themes: community, including not feeling alone, solidarity, and recognizing similarities despite differences; appreciation, including feeling heard and seen and leaving with a positive feeling; and holistic understanding, including learning from others’ experiences and gaining different perspectives. When asked about the most important next step, participants most frequently emphasized continued opportunities for exchange, including further forums and stronger networking.

During the plenary feedback, facilitators presented their groups’ views that the combination of external facilitation and the co-hosting by Herzklopfen e.V. supported open discussion and made it easier to express concerns about day-to-day strain, uncertainty, and unmet support needs.

The process evaluation also suggested a potential dual function of the forum: while its primary purpose was to contextualize PROM findings, participants’ responses indicated that the participatory format also created opportunities for connection, mutual recognition, and networking among families.

## Discussion

This paper describes an adaptable, PROM-anchored participatory interpretation forum that was embedded within a longitudinal pediatric HRQoL study and co-developed with a patient organization. Its main contribution is methodological: rather than using PROM data only as a feedback product at the end of the study, the forum turned interim results into a shared interpretive resource that families, clinicians, and the patient organization could revisit and translate into action.

### IKT and co-production

A key strength of this approach is the integration of several iKT mechanisms within a single process: shared governance between clinical and patient/family advocacy partners, the participatory contextualization of interim PROM trends, and the co-designed dissemination of findings through the “Quality-of-Life Tree”. The format moved beyond consultation and closer to co-production because the patient organization both received results and were able to actively modify recruitment, hosting, interpretation, and the return of findings, thereby promoting a more balanced power dynamic between research and community perspectives [2, 4, 5, 22].

Our forum also enabled methodological responsiveness to community feedback. While the participatory contextualization suggested that the PedsQL CM captures important disease-specific and functional CHD aspects, collaborative discussions revealed that families also interpret HRQoL through a psychosocial and transitional lens. Because key aspects such as participation and the significance of transitions are not fully captured by the current questionnaire, future work may consider whether additional instruments such as the Congenital Heart Disease Specific Inventory [23] could complement the PedsQL CM in capturing lived experience more comprehensively.

### Why it matters for PROM implementation and care

Our forum adds to the PROM implementation literature by showing how aggregate PROM results can become usable before the end of data collection. A recurring challenge in PROM work is that scores can remain detached from the experiences they are meant to represent. Our results illustrate why participatory interpretation matters in practice: families did not simply confirm that the HRQoL domains were relevant, they specified what those domains meant in their everyday lives. This is important because PROM trends gain explanatory and clinical value only when they are embedded within family routines, expectations, and coordination demands [12].

Our approach also sharpened the prioritization of already well-recognized needs by situating them within family routines, transition points, and coordination demands. The action list and plenary discussion pointed toward transition-sensitive support, child-friendly explanations, identifiable points of contact, and better coordination across clinic, school, and community services.

Importantly, the priorities and interpretations generated in this forum should not be understood as definitive interpretations of the PROM findings. Families may draw on different clinical experiences, care trajectories, and expectations when making sense of the same quantitative patterns, and priorities are therefore likely to be context-dependent. Hence, the present study demonstrates feasibility and procedural transparency rather than context-free replication across settings. Repeating the process with different groups of families may consequently generate different, rather than merely confirmatory, priorities and practice-oriented actions. In this sense, transferring our forum model would serve not only to assess whether the process can be implemented across contexts, but also to explore the range of meanings and priorities that may emerge from different family perspectives.

### Dissemination as part of the method

Exclusively end-of-study dissemination risks creating a bottleneck between data collection and use, whereas an embedded format can support earlier sense-making and swifter follow-up. In this way, our forum narrowed the distance between measurement and clarification of meaning and operationalized participatory interpretation as a bounded, transferable study component. The “Quality-of-Life Tree” figure provided a condensed dissemination format for our forum’s interpretive outputs. It translated the shared understanding into a family-facing visual summary that separated foundational quality-of-life elements, thereby stabilizing supports, shifting challenges, and future-oriented subsequent steps. In so doing, it closed the feedback loop between PROM measurement, participatory interpretation, and the family-oriented return of results. The choice of a tree metaphor was deliberate: it was intended to support returning findings in a form that felt constructive, growth-oriented, and agency-enhancing rather than deficit-based. Participatory research must move beyond simply extracting data and insights from families; it should share accessible findings with them as well. This ensures genuine reciprocity, reflecting the CBPR principles of transparent and meaningful results sharing.

### Limitations

Several limitations should be acknowledged. First, the forum drew on a self-selected and relatively engaged sample, likely including families with stronger ties to the patient organization or clinic network, which may have shaped both the issues raised and the priorities identified. Although invitations were also distributed through clinic channels and participation did not require organizational membership, residual selection bias cannot be excluded. Given the composition of the forum, parent and caregiver perspectives may also have been more strongly represented than those of adolescents, and the online format may have further shaped opportunities for participation.

Second, the facilitation structure may have influenced the interactional context. Although an independent moderator facilitated the plenary sessions and small-group facilitators received specific preparation in group processes and participatory facilitation, the breakout discussions were largely facilitated by professionals from the clinical and psychosocial setting. Facilitators did not contribute their own substantive positions but represented the views expressed within their groups. Nevertheless, professional roles may have shaped the interactional context in ways that cannot be fully assessed retrospectively. Dominant voices and social desirability effects cannot therefore be excluded. Although participants described the discussion environment as open and supportive, these perceptions were expressed within the forum itself and were not assessed through an independent evaluation of participation patterns or power dynamics.

Third, the rapid note-based analysis increased feasibility and timeliness but reduced analytic granularity compared with full transcript-based analysis. The approach was intentionally oriented toward identifying breadth and actionable directions rather than providing in-depth qualitative or phenomenological analysis.

Finally, the format was implemented in a single condition-specific context and in partnership with a well-established patient organization, which may limit its transferability to settings without a similar relational or organizational infrastructure. Importantly, transferability of the process does not imply that the same substantive priorities would emerge in other groups. Families with different clinical experiences, care trajectories, or levels of engagement may interpret the same PROM patterns differently and prioritize different actions. The findings should therefore be understood as context-specific participatory interpretations rather than as definitive validation of the quantitative findings.

### Implications and future directions

The forum also informed several subsequent activities. The longitudinal study was complemented by a qualitative cross-sectional component to explore individual experiences underlying the questionnaire findings in greater depth, particularly regarding treatment-related anxiety. The forum also highlighted the potential value of similar participatory formats with adolescents and young adults with CHD, including those with adult congenital heart disease, to strengthen networking and identify age-specific needs. Building on the forum discussions, a “Kardio-Kompass” was developed in collaboration with Herzklopfen e.V. to provide families with structured information on psychosocial and educational services, support offered by the patient organization, basic information on the cardiovascular system, and support related to transitions such as school entry. Contact was also established with the Bundesverband Herzkranke Kinder e.V. (BVHK) to share experiences from the forum and explore opportunities for disseminating relevant support approaches across patient organizations.

Future work should examine whether and how such forums can be embedded more routinely into longitudinal PROM workflows, including whether repeated forums at different stages of data collection can incorporate changing priorities and experiences over time. Comparative work across diagnoses, age groups, care settings, and levels of stakeholder involvement would help clarify not only which contextual features are necessary for the format to work well and which are adaptable, but also how different family perspectives may shape the priorities and actions emerging from the same PROM findings. It would also be worthwhile to study the format’s resource implications including the required time, staffing, and facilitation effort, as well as the cost-effectiveness in relation to the implementation gains it may generate. An additional methodological consideration for teams seeking to adapt this format concerns the overlap between forum participants and participants in the underlying longitudinal study. In the present project, this overlap was intentional, as the forum aimed to contextualize PROM findings with the experiences of the population from which the data originated. However, researchers considering similar approaches should be aware that feeding back interim findings to study participants may itself influence subsequent responses, engagement with data collection, or participants’ interpretation of their own experiences. Future work should therefore examine how different degrees of participant overlap affect both the participatory process and the longitudinal data collection it accompanies. Finally, future studies should examine whether the participatory interpretation process supports sustained engagement, implementation of agreed actions, and more beneficial feedback for families and clinicians.

### Closing perspective

Overall, this paper shows that PROMs become more actionable when they are not only measured and reported, but also discussed in a structured participatory setting that makes interpretation visible and shared. The value of PROM data lies not just in the score itself, but in the collective work of translating the score into meaning, priorities, and subsequent steps. For methods research, that makes our forum a useful model of how measurement, lived experience, and dissemination can be integrated into a single workflow. More broadly, it illustrates that participatory and co-produced research can strengthen both methodological validity and practical relevance.

## Supplementary Information

Below is the link to the electronic supplementary material.

Supplementary Material 1

## Data Availability

No datasets were generated or analysed during the current study.

## Abbreviations

CBPR: Community-based participatory research
CHD: Congenital heart disease
GRIPP2: Guidance for reporting involvement of patients and the public, version 2
HRQoL: Health-related quality of life
iKT: integrated Knowledge Translation
PedsQL CM: Pediatric quality of life inventory™ cardiac module
PPI: Patient and public involvement
PROM(s): Patient-reported outcome measure(s)
