# Supplementary material for: Translating scores into meaning: a co-produced forum for patient-reported outcome measure interpretation in pediatric congenital heart disease
Source: Res Involv Engagem. 2026 Sep 28;12:158. doi: 10.1186/s40900-026-00981-7 (PMC13621704; doi:10.1186/s40900-026-00981-7)
Supplement: Supplementary file 1 [file 40900_2026_981_MOESM1_ESM.docx]

# Additional File 1 - GRIPP2 short form

## Study Characteristics

| **Project/study name** | PROM-anchored participatory forum for longitudinal pediatric HRQoL monitoring in children and adolescents with congenital heart disease |
| --- | --- |
| **Researcher lead name** | Dr. Christoph Zürn and Jana Willems |
| **PPI lead/coordinator name** | Petra Huth, chairperson of Herzklopfen e.V. (in close coordination with the whole board) |
| **PPI groups utilized (if relevant)** | Herzklopfen e.V. as parent-led PPI partner; families of children and adolescents with congenital heart disease; clinical staff and research team members participating as facilitators and co-interpreters |
| **Number of public contributors involved in the project** | 20 families; 27 participants in total including 6 adolescents and 21 parents |
| **Diversity of public contributors used in the project** | Participants included children/adolescents with congenital heart disease and their caregivers. Recruitment was conducted through both the patient organization and hospital channels, allowing participation from families with different levels of connection to the patient organization and clinical care. The forum included both young people and caregivers across a broad age range. However, the sample was likely more engaged than the broader target population, and no systematic sampling strategy was used to ensure representation across sociodemographic or clinical characteristics. |

## Official GRIPP2-SF Checklist Items

### Aim

To co-design, co-deliver, and co-interpret a PROM-anchored participatory forum that contextualized interim questionnaire results and co-produced actionable next steps for clinical practice and service improvement.

### Methods

PPI was embedded through a partnership with Herzklopfen e.V., a parent-led organization for families of children with congenital heart disease. The organization co-developed the forum concept through regular preparatory meetings with the chairperson, research team, external moderator, and small-group facilitators. The design was revised iteratively at board level, and the organization co-designed recruitment materials, communication channels, hosting arrangements, and registration management. During the forum, Herzklopfen e.V. co-hosted the event and contributed to interpretation and prioritization. After the forum, a structured debrief and joint analysis session supported validation of draft interpretation memos and planning of next steps.

### Study results (impact of PPI)

PPI improved the accessibility of the forum by shaping plain-language presentation and enabling recruitment through trusted channels. It also strengthened the contextual interpretation of PROM domains by incorporating family experience specifically within the discussion of quantitative results. The outputs gained legitimacy through organizational involvement, and the forum produced a shared action list and family-facing dissemination figure. A potential negative effect was that recruitment through a parent-led organization may have favored more engaged families and therefore limited representativeness.

### Discussion and conclusions

PPI influenced the project at multiple levels: design, recruitment, delivery, interpretation, dissemination, and follow-up planning. Overall, the partnership moved the process beyond consultation towards co-production. This strengthened the interpretive validity of the outputs and increased the likelihood that the findings would inform implementation and practice. At the same time, the collaborative model required substantial coordination and may not be equally feasible in settings without a strong patient organization partner.

### Reflections/critical perspective

The partnership with Herzklopfen e.V. worked well because it created trust, improved communication, and supported meaningful co-interpretation. However, close organizational partnership can also introduce alignment bias and may overrepresent more active or resourceful families. Future iterations should actively include underrepresented perspectives, consider additional recruitment channels, and examine how different PPI configurations affect interpretation and downstream implementation.
